# Development and optimization of AI algorithms for wrist fracture detection in children using a freely available dataset

**DOI:** 10.3389/fped.2023.1291804

**Published:** 2023-12-21

**Authors:** Tristan Till, Sebastian Tschauner, Georg Singer, Klaus Lichtenegger, Holger Till

**Affiliations:** ^1^ Department of Applied Computer Sciences, FH JOANNEUM - University of Applied Sciences, Graz, Austria; ^2^ Division of Pediatric Radiology, Department of Radiology, Medical University of Graz, Graz, Austria; ^3^ Department of Pediatric and Adolescent Surgery, Medical University of Graz, Graz, Austria

**Keywords:** artificial intelligence, machine learning, children, trauma, wrist, fracture, training, YOLO

## Abstract

**Introduction:**

In the field of pediatric trauma computer-aided detection (CADe) and computer-aided diagnosis (CADx) systems have emerged offering a promising avenue for improved patient care. Especially children with wrist fractures may benefit from machine learning (ML) solutions, since some of these lesions may be overlooked on conventional X-ray due to minimal compression without dislocation or mistaken for cartilaginous growth plates. In this article, we describe the development and optimization of AI algorithms for wrist fracture detection in children.

**Methods:**

A team of IT-specialists, pediatric radiologists and pediatric surgeons used the freely available GRAZPEDWRI-DX dataset containing annotated pediatric trauma wrist radiographs of 6,091 patients, a total number of 10,643 studies (20,327 images). First, a basic object detection model, a You Only Look Once object detector of the seventh generation (YOLOv7) was trained and tested on these data. Then, team decisions were taken to adjust data preparation, image sizes used for training and testing, and configuration of the detection model. Furthermore, we investigated each of these models using an Explainable Artificial Intelligence (XAI) method called Gradient Class Activation Mapping (Grad-CAM). This method visualizes where a model directs its attention to before classifying and regressing a certain class through saliency maps.

**Results:**

Mean average precision (mAP) improved when applying optimizations pre-processing the dataset images (maximum increases of +25.51% mAP@0.5 and +39.78% mAP@[0.5:0.95]), as well as the object detection model itself (maximum increases of +13.36% mAP@0.5 and +27.01% mAP@[0.5:0.95]). Generally, when analyzing the resulting models using XAI methods, higher scoring model variations in terms of mAP paid more attention to broader regions of the image, prioritizing detection accuracy over precision compared to the less accurate models.

**Discussion:**

This paper supports the implementation of ML solutions for pediatric trauma care. Optimization of a large X-ray dataset and the YOLOv7 model improve the model’s ability to detect objects and provide valid diagnostic support to health care specialists. Such optimization protocols must be understood and advocated, before comparing ML performances against health care specialists.

## 1. Introduction

In pediatric medicine, artificial intelligence (AI) has found valuable applications to support healthcare professionals in tasks such as automating processes, retrieving information and providing decision support (1, 2). AI-based solutions may be extremely helpful, when a radiological question occurs frequently, so that considerably big data sets are available, but specific challenges still remain and require long-term experience (3). This constellation certainly applies to pediatric wrist fractures, since some of these lesions may be overlooked on conventional X-ray due to minimal compression without dislocation or mistaken for cartilaginous growth plates (4).

The plethora of terms used within the field of AI still causes difficulties or even confusion. Basically, the broad umbrella term is artificial intelligence (AI). Machine learning (ML) is one area of AI. Deep Learning (DL) is part of ML and makes use of artificial neural networks (ANN). A neural network is an AI variant that teaches computers to process data in a way that was originally inspired by the human brain and uses interconnected nodes or neurons, usually in a layered structure. Image classification regularly relies on convolutional neural networks (CNN), a special type of ANN (5). The main idea of CNNs, described well e.g. in Ch. 14 of Murphy (6), is to learn small filters, which (similar to those used in image processing) recognize certain features like horizontal or vertical edges. These features are combined, using higher-order filters on higher hierarchy levels (i.e. in later layers of the network), to identify first basic geometric structures (like rectangles, arcs and circles) and later on objects composed of these basic objects.

This holds true also for the “You Only Look Once” (YOLO) object detectors, first published in 2015/16, which has received several upgrades to newer versions (7, 8).

Regarding automated pediatric wrist fracture detection, computer-aided detection (CADe) and computer-aided diagnosis (CADx) systems based on big data sets contribute to the detection of rarities within the pool of available data (9). However, ensuring complete explainability and trustworthiness behind AI models remains a challenging endeavor as long as specific IT knowledge remains limited in healthcare professionals, both in general but also regarding the presented fracture detection problem.

Neural networks, especially deep neural networks, often operate in ways that are intricate and difficult for humans to comprehend (10). Consequently, Explainable Artificial Intelligence methods are employed to present machine learning outcomes in an understandable manner. These outcomes can take the form of visualizations, textual explanations, or examples (11, 12). A noteworthy method is Gradient-weighted class activation mapping (Grad-CAM), which adapts traditional Class Activation Mapping (CAM) in a model-agnostic manner, allowing for more than global average pooling. Additionally, Guided Grad-CAM acts as a hybrid technique, combining Grad-CAM and guided backpropagation through element-wise multiplication. This approach yields higher-resolution visualizations that are class-specific and discriminatory (11, 13).

The superordinate goal of this article, which extends and deepens the analysis presented in Till et al. (14), is to describe the development and optimization of AI algorithms for wrist fracture detection in children using a freely available dataset. It highlights the steps that lead to improved performance and the role that each of the medical and technical players must play to achieve this.

## 2. Materials and methods

This study assessed the influence of several variations and settings of the seventh generation YOLO (8) model (YOLOv7), a state-of-the-art object detector.

### 2.1. Dataset

The experiments were based on the openly available pediatric wrist trauma dataset “GRAZPEDWRI-DX” (15), published by the Division of Pediatric Radiology, Department of Radiology, Medical University of Graz in 2022. This dataset contains annotated pediatric trauma wrist radiographs of 6,091 patients, and a total number of 10,643 studies (20,327 images). All of the provided studies were included.

### 2.2. Model training

During training, the model learns by repeatedly making predictions, measuring the error between the prediction and the expected result, and adjusting internal parameters accordingly. Usually, some version of gradient descent is used for this, i.e. the process can be illustrated as “moving downhill” on an abstract landscape generated by the prediction error. The learning rate governs how fast this “movement” is, and often also some version of momentum is incorporated (16).

Care has to be taken not to *overfit* the model, i.e. not to have the model learn irrelevant details of the training data and losing its ability to generalize (17). This is usually achieved by performing a train-test-split, i.e. training the model only on some portion of the data, called the *training set*, and evaluating the prediction error also on the rest, called the *test set* or *validation set*. The ideal ratio of these splits vary depending on the data, with 80% typically being used for larger datasets (18). When the error decreases on the training set, but systematically increases on the validation set, this is a strong indication for the onset of overfitting (17).

When aiming for better model performance, this can be achieved, on the one hand, by enhancing the quality or amount of data fed into the network during training. In this study, we have varied image sizes and preprocessing procedures (19). On the other hand, directly altering the object detection model, like using different architectures (8) or adjusting hyperparameters during training (20), influence detection mean average precision as well. In this study, we agreed upon that the initial learning rate and momentum play a crucial role for optimizing clinical decision-making in pediatric wrist trauma care, which made them part of the analysis during experiments.

Models were trained on a Virtual Machine with 15GB RAM, 100GB hard drive storage, 4 CPU cores as well as a dedicated NVIDIA GPU. Training was performed using the code published with the original YOLOv7 paper (8) executing the scripts in the Command Line. Model training duration ranged from 12 to 24 h for all variations. However, it was agreed upon that differences in training time will not be taken into consideration for the upcoming analysis.

#### 2.2.1. Hyperparameters

**Figure 1:**
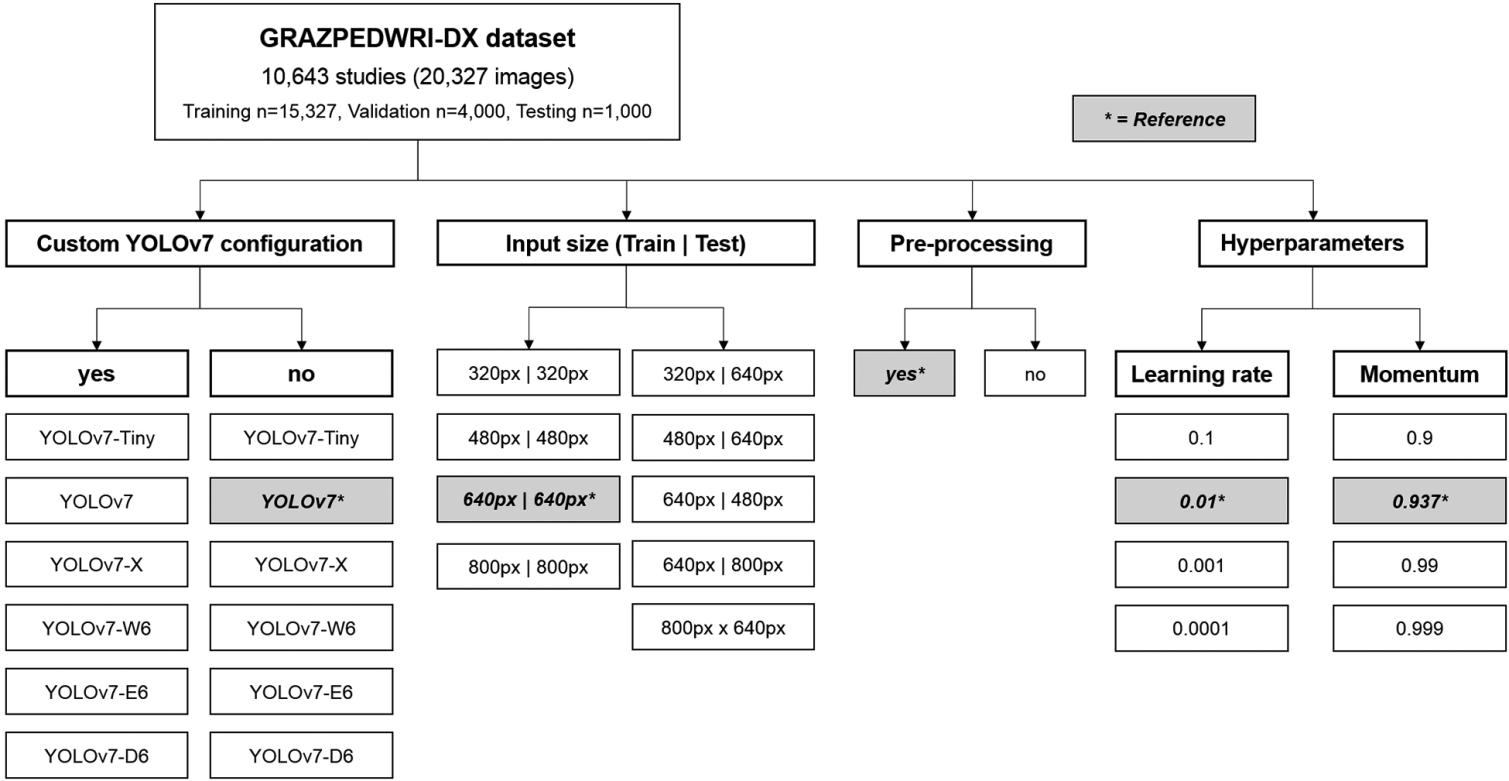
Flowchart depicting the variations of different YOLOv7 models, input sizes, pre-processing and hyperparameters assessed. The reference values are marked by an asterisk.

In machine learning, hyperparameters (like learning rate, influence of momentum or batch size) are different to standard model parameters, as they stay constant throughout the training process and are not contained in the final model. They greatly influence the speed of the training process and determine how the model is adjusted when new information becomes available. Thus, hyperparameter search often constitutes a major challenge in designing machine learning models. We decided to test 3 alternative learning rates and momentum settings in this manuscript (Figure 1).

### 2.3. Explainable artificial intelligence

In many disciplines, including healthcare, but also for example finance, it is not sufficient for an AI model to just have a good predictive performance. For such models, it is also regarded as vitally important to be able to *explain* for which reasons a prediction has been made. Because of this, weaker, but transparent models (like linear scores or decision trees) are often preferred to more powerful but largely opaque models like neural networks.

For example, CNNs have achieved remarkable performance on many image classification tasks, but to explain *why* the network has come to a decision is no trivial task.

However, sacrificing predictive power in favor of transparency is considered as highly problematic as well, and considerable effort has been invested to even make complex models more transparent. The resulting field of Explainable Artificial Intelligence (XAI) encompasses both model-agnostic approaches, which can be used with any ML model, and approaches that are only applicable to specific ML methods.

**Figure 2:**
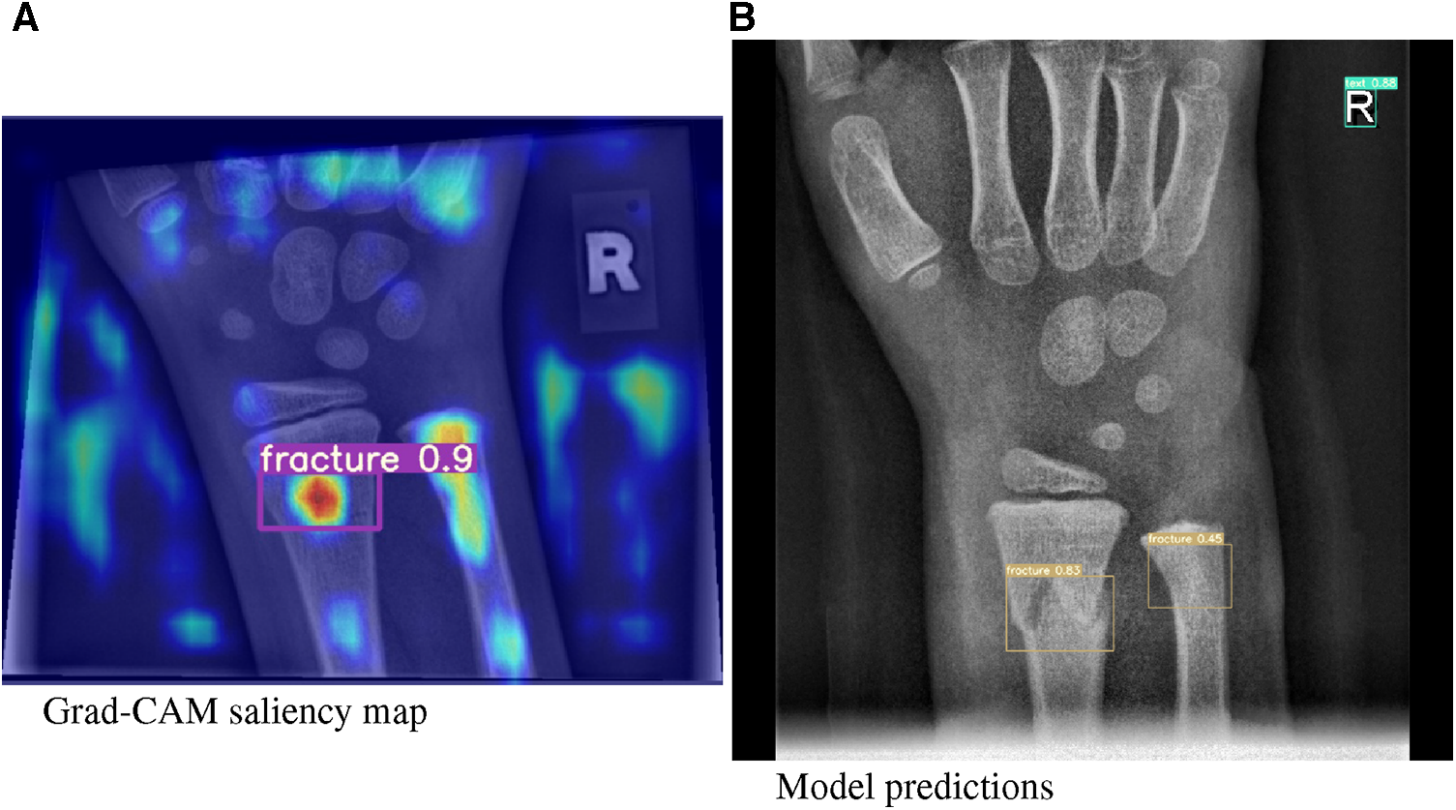
(**A**) Grad-CAM saliency maps for image 1430_0611557520_01_WRI-R1_M008.png. (**B**) Model predictions for image 1779_0477729910_01_WRI-R1_M004.png by means of a bounding box.

In this article, we investigated our models using a XAI method called Gradient Class Activation Mapping (Grad-CAM) (13), ass exemplarily demonstrated in Figure 2, which is specifically designed for the analysis of image recognition processes. Through saliency maps, this method visualizes where a model directs its attention to in the process of predicting a certain class.

### 2.4. Model performance and metrics

It is crucial to analyse the performance of an AI model using adequate metrics and to provide the results in a comprehensible way. A number of different methods are available to accomplish these tasks. These have evolved over time and, depending on the application area, are now relatively standardised.

With regard to the recognition of objects, the most commonly used metrics are

• *IoU (Intersection over Union)*. An IoU of 1 means a perfect match of the AI prediction and the ground truth. An IoU of 0 corresponds to no overlap of the labels.
• *Precision*: Number of true positives (TP) over sum of true positives (TP) and false positives (FP).
• *Recall*: Number of true positives (TP) over sum of true positives (TP) and false negatives (FN).
• *Average precision (AP)* is the area under a precision-recall curve for an object of interest.
• *Mean average precision (mAP)* is the AP over all tested classes. Typically, the mAP is calculated for an IoU threshold of 0.5 (50% = mAP@[0.5]) and averaged between 0.5 and 0.95 with 5% steps (mAP@[0.5:0.95]) (21).

## 3. Results

**Figure 3:**
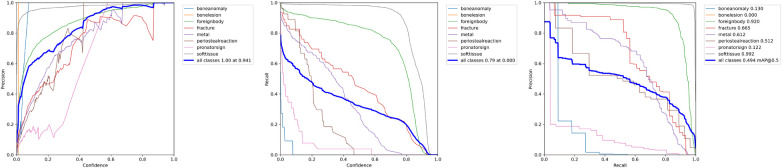
P-Curve, R-Curve and PR-Curve for the reference model and settings.

In comparison to the reference performance depicted in Figure 3, performance metrics benefited from employing more complex YOLOv7 architectures, which introduce more layers, parameters and gradients into the neural network. The increases in performance are displayed Table 1. However, training larger models did not automatically produce more precise or accurate models. mAP increases reached a maximum of +13.16% mAP@0.5 and +24.09% mAP@[0.5:0.95], respectively for the YOLOv7-X configuration, which is the 4th largest YOLOv7 variation analysed.

**Table 1:** List of the best performing settings in every group of methods.

Method	Var	Precision	Recall	mAP@0.5	mAP@[0.5:0.95]	Inference time (ms)
Baseline	Default	0.67	0.497	0.494	0.274	12.5
Architecture	YOLOv7-Tiny	0.774	0.536	0.544	0.326	11
Configuration	YOLOv7-X	0.778	0.549	0.559	0.34	13
Image sizes	800×640	0.551	0.783	0.62	0.383	12.4
Preprocessing	No Edit	0.737	0.623	0.64	0.385	11.6
Learning rate	0.001	0.691	0.609	0.554	0.348	12.4
Momentum	0.99	0.636	0.555	0.528	0.321	12.6

**Figure 4:**
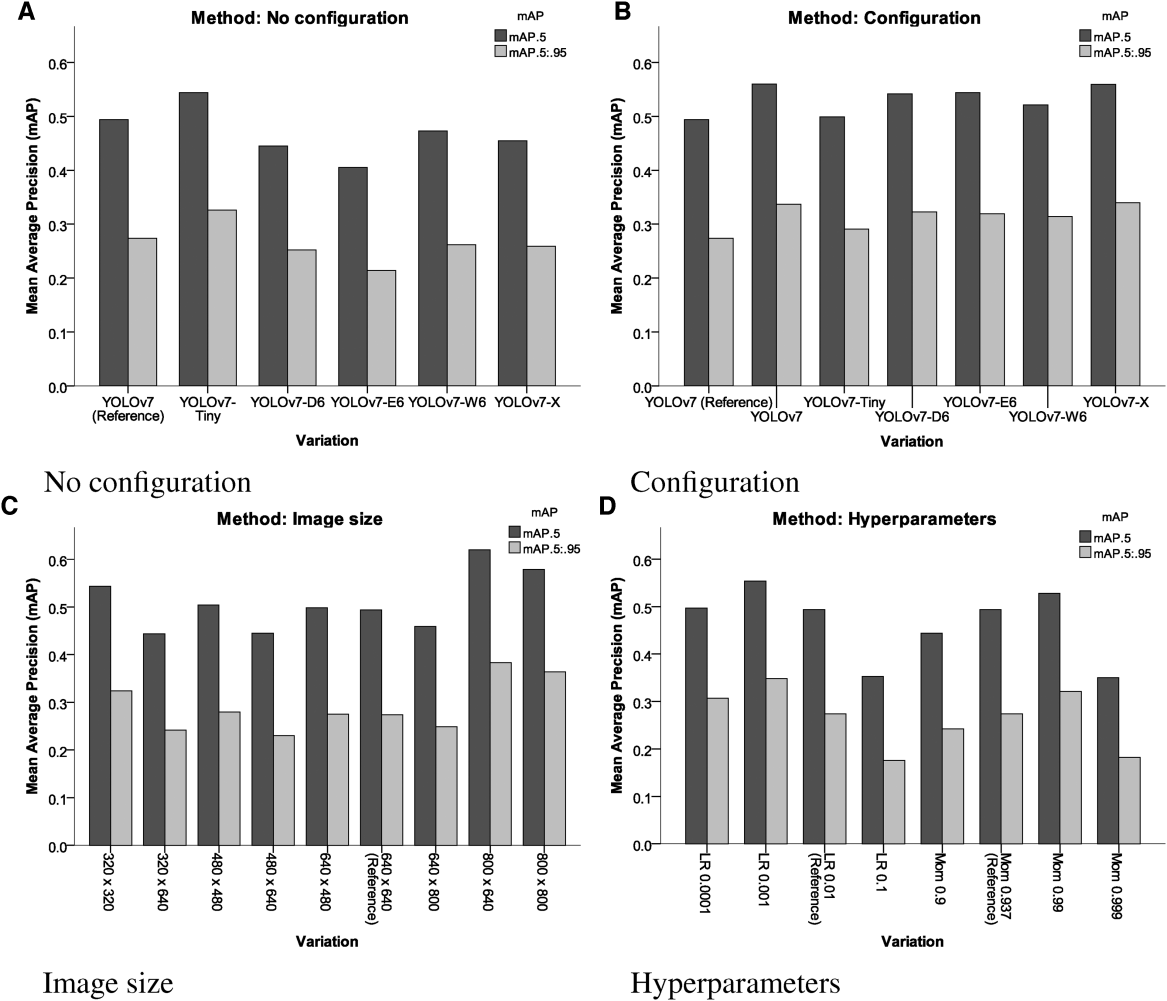
Results of the different variations of different YOLOv7 models, input sizes, pre-processing and hyperparameters assessed. LR, learning rate; Mom, momentum. (**A**) No configuration, (**B**) configuration, (**C**) image size and (**D**) hyperparameters.

Moreover, similar results could be achieved by decreasing the learning rate to 0.001 (+12.15 mAP@0.5, +27.01 mAP@[0.5:0.95]), or increasing momentum to 0.99 (+6.88 mAP@0.5, +17.15 mAP@[0.5:0.95]) for a model of equal size. mAP was improved when omitting image preprocessing procedures (+17.65% mAP@0.5, +18.10% mAP@[0.5:0.95]) and increasing image sizes during training (+25.51% mAP@0.5, +39.78% mAP@[0.5:0.95]) (compare Figure 4).

Generally, when analysing the resulting models using XAI methods, higher scoring models in terms of mAP paid more attention to broader regions of the image, prioritizing detection accuracy over precision compared to the less accurate models.

**Figure 5:**
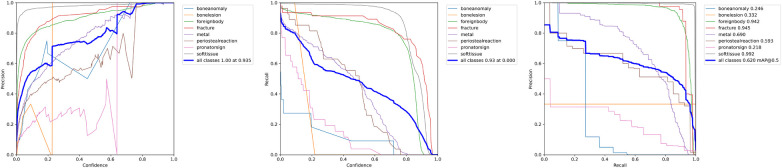
P-Curve, R-Curve and PR-Curve for the model trained with an increased image size of 800×640 pixel.

The results in Table 1 indicate that the most important parameters are the avoidance of pre-processing procedures and a high image size (Figure 5). These two parameters more important than choosing different YOLOv7 architectures.

## 4. Discussion

Wrist fractures are the most common fractures diagnosed in children and adolescents (22). While some fractures of the distal forearm do not even require conventional X-ray to confirm the diagnosis because of an obvious clinical malalignment, minor fractures in young children may easily be overlooked, because they cause only minimal radiological signs of bony compressions. Furthermore, cartilaginous growth zones may mimic or deceive actual fractures (23). Thus, specific medical training as a pediatric radiologist or pediatric surgeon is required to be familiar with age-specific details of the growing skeleton. Nevertheless, all forms of pediatric wrist fractures require adequate diagnosis and therapy to minimise potential subsequent growth disturbances (24). Paediatric trauma radiographs are often interpreted by emergency physicians and adult radiologists, sometimes without specialization in pediatric X-rays or back-up by experienced pediatric radiologists. Even in developed countries, shortages of radiologists were reported, posing a risk to pediatric patient care. In some parts of the world, access to pediatric radiologists is considerably restricted, if not unavailable (25).

Thus, particularly in the field of pediatric radiology, computer-aided detection (CADe) and computer-aided diagnosis (CADx) systems could contribute to the interpretation of patient data and scans (9). The roots of CAD systems trace back to the late 1950s when biomedical researchers first explored the potential of expert systems in medicine. These early endeavors involved computer programs that took patient data as input and generated diagnostic outputs. As technology progressed, these initial approaches evolved and were refined, incorporating AI and specialized algorithms to enhance predictive capabilities (26). In medical imaging there are several approaches to help physicians making a correct diagnosis. In addition to simple classification, i.e. predicting, whether a feature is present or not in the respective image, methods for localizing pathologies have also proven successful. A common approach involves identifying and delineating objects through bounding boxes (boxes around a predicted object), or image segmentation (masks or polygonal shapes around a predicted object) (27).

Medicine, and especially imaging are in the midst of a transformation towards AI, and AI algorithms are playing an increasingly important role in diagnostics (28). Some solutions are visible to the user in terms of computer assistance or interaction, while others work in the background to make radiological examinations even better (29). The clinical relevance of the available AI solutions is growing steadily. In a few years’ time, the use of optimised assistance solutions - such as the one presented in this manuscript - could become indispensable. It is not unlikely that CAD systems for recognising fractures or pathologies might also be available as open source solutions, community projects or educational resources in the future (30), alongside commercial products. Language models and generative AI have recently shown us this possibility (31). Seamless integration of useful AI tools will play a decisive role in their acceptance.

Given the complexity of the challenges in this domain, deep learning algorithms have risen to prominence, surpassing traditional neural networks in various aspects (32). In some cases, deep learning algorithms demonstrate comparable or even superior performance to human medical professionals (33). However, the increasing complexity of these models, the reliance on high-quality data, and concerns related to explainability and ethics have posed hurdles to their full-scale practical implementation. Any technology in the medical field must embody simplicity, safety, and trustworthiness to be of true benefit to healthcare practitioners (10).

In this study, we delved into the examination of a YOLOv7 object detector with a focus on detecting pediatric wrist fractures. The dataset and baseline parameters used here closely followed a study by Nagy et al. (15), ensuring a realistic scope and resource context. Accordingly, this paper undertook an ablation study, established a baseline model, and subsequently presents the research findings (10).

Overall, using configuration files improved model performance, except for YOLOv7-Tiny. Different architectures scaled similarly to or above YOLOv7 performed similarly well. Yet, configuration adjustments did not consistently enhance all models. Further investigation is needed to understand their relationship. Adjusting learning rate and momentum had significant impacts on performance. Lower learning rates led to better precision, compensating for imbalanced classes. An initial learning rate of 0.001 worked best. A momentum of 0.99 yielded optimal results overall.

Experimenting with image sizes during testing and training resulted in more variable outcomes. Training on larger images and testing on smaller ones yielded better results for all classes. Testing on larger images reduced performance, similar to omitting configurations. Unedited data improved average precision and reduced inference time, especially for fractures. Adjusting image size for training to 800×800 produced the best results overall, benefiting average precision. Smaller image sizes helped object detection in general, but were less effective for fractures.

**Figure 6:**
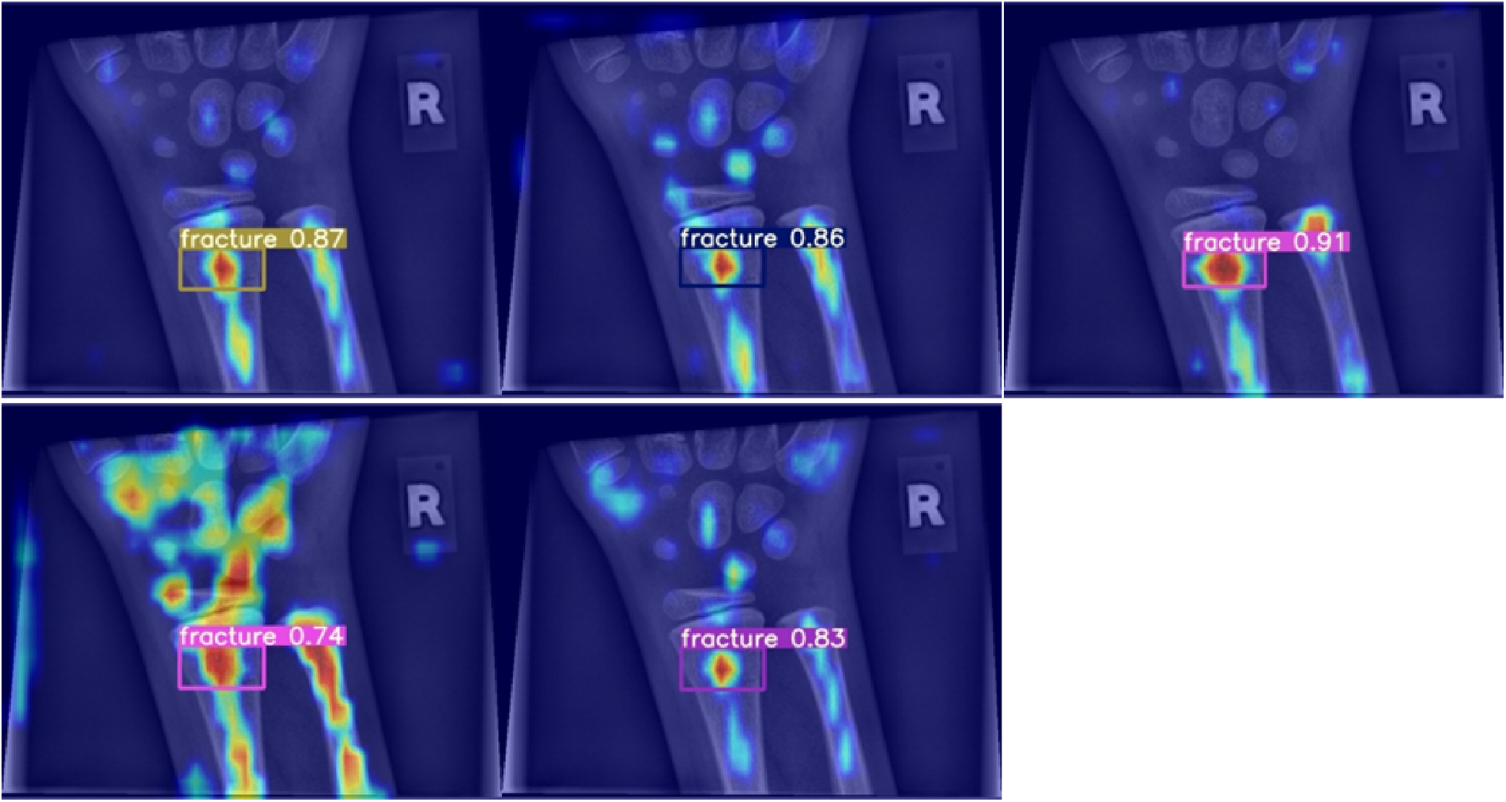
Grad-CAM for models performing worse than the baseline in terms of mean average precision.

**Figure 7:**
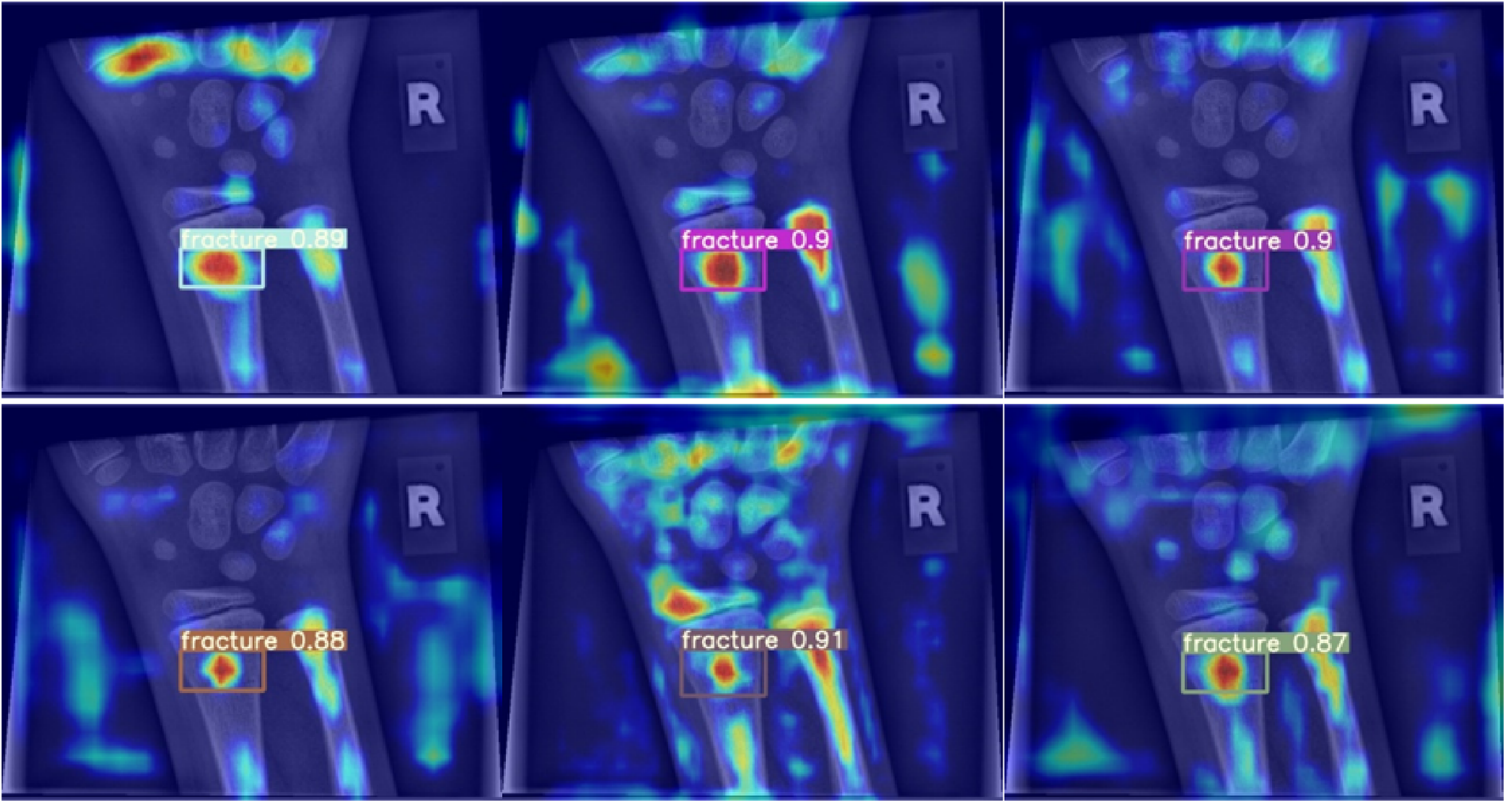
Grad-CAM for models performing better than the baseline in terms of mean average precision.

When analysed with Grad-CAM, models that outperformed the reference model in terms of mean average precision tended to generate noisier saliency maps, directing attention to the predicted object areas. Worse models produced more focused maps around actual fractures (compare Figure 6). Higher scoring models performed better due to their ability to minimize the amount of false negatives during detection, rather than maximizing IoU scores during regression, thus valuing detection accuracy over detection precision (Figure 7).

Some limitations of the manuscript need to be mentioned: We analysed the seventh iteration of the YOLO algorithm for object detection, while, in the meantime, a newer version has been released. It has to be understood that there might be small improvements in detection performance. Moreover, we did not compare the model results with medical experts. The reason is that we wanted to highlight the various technical possibilities to improve fracture detection performance during model development. We did not include statistical analyses in this manuscript, primarily because a comparison of model performances on image or bounding box levels with a test set of n=1,000 always result in p values below 0.05, even in minor group differences. On the other hand, a statistical comparison of the mAP values are not possible, because of the single metric value for each group. Thus, the presented differences between the individual architectures and settings need to be judged for their clinical and technical relevance. We were not able to test higher image sizes above 800×800 pixels due to computational restraints. The results showed a dependency of image size with model performance, and higher image sizes might lead to further improvements in model performances.

## 5. Conclusions

Adjusting the dataset and image size had the most significant impact on average precision during testing. Sharpening and contrast enhancement hindered feature learning, and larger image sizes improved results despite testing on smaller images. Larger architectures did not always guarantee better performance. Hyperparameter adjustments also influenced results, with customized hyperparameters improving average precision by up to 27.01%. Grad-CAM analysis highlighted model strengths and weaknesses. This study contributes to understanding machine learning’s potential in pediatric healthcare, emphasizing the importance of data and configuration considerations. Such optimization protocols must be understood and advocated, before comparing ML performances against health care specialists.

## Data Availability

A publicly available dataset was used in this study, accessible under: https://doi.org/10.1038/s41597-022-01328-z
